# Appropriate Use of Cardiac Stress Testing with Imaging: A Systematic Review and Meta-Analysis

**DOI:** 10.1371/journal.pone.0161153

**Published:** 2016-08-18

**Authors:** Joseph A. Ladapo, Saul Blecker, Michael O'Donnell, Saahil A. Jumkhawala, Pamela S. Douglas

**Affiliations:** 1 Division of General Internal Medicine and Health Services Research, David Geffen School of Medicine, University of California Los Angeles, Los Angeles, CA, United States of America; 2 Department of Population Health and Medicine, New York University School of Medicine, New York, NY, United States of America; 3 New York University School of Medicine, New York, NY, United States of America; 4 New York University, New York, NY, United States of America; 5 Department of Medicine, Duke University School of Medicine, Durham, NC, United States of America; Shenzhen institutes of advanced technology, CHINA

## Abstract

**Background:**

Appropriate use criteria (AUC) for cardiac stress tests address concerns about utilization growth and patient safety. We systematically reviewed studies of appropriateness, including within physician specialties; evaluated trends over time and in response to AUC updates; and characterized leading indications for inappropriate/rarely appropriate testing.

**Methods:**

We searched PubMed (2005–2015) for English-language articles reporting stress echocardiography or myocardial perfusion imaging (MPI) appropriateness. Data were pooled using random-effects meta-analysis and meta-regression.

**Results:**

Thirty-four publications of 41,578 patients were included, primarily from academic centers. Stress echocardiography appropriate testing rates were 53.0% (95% CI, 45.3%–60.7%) and 50.9% (42.6%–59.2%) and inappropriate/rarely appropriate rates were 19.1% (11.4%–26.8%) and 28.4% (23.9%–32.8%) using 2008 and 2011 AUC, respectively. Stress MPI appropriate testing rates were 71.1% (64.5%–77.7%) and 72.0% (67.6%–76.3%) and inappropriate/rarely appropriate rates were 10.7% (7.2%–14.2%) and 15.7% (12.4%–19.1%) using 2005 and 2009 AUC, respectively. There was no significant temporal trend toward rising rates of appropriateness for stress echocardiography or MPI. Unclassified stress echocardiograms fell by 79% (p = 0.04) with updated AUC. There were no differences between cardiac specialists and internists.

**Conclusions:**

Rates of appropriate use tend to be lower for stress echocardiography compared to MPI, and updated AUC reduced unclassified stress echocardiograms. There is no conclusive evidence that AUC improved appropriate use over time. Further research is needed to determine if integration of appropriateness guidelines in academic and community settings is an effective approach to optimizing inappropriate/rarely appropriate use of stress testing and its associated costs and patient harms.

## Introduction

Cardiac imaging has advanced physicians’ ability to diagnose and treat a variety of diseases, but rapid growth in the utilization and cost of imaging technology has spurred public and private insurers to scrutinize its use and construct policies aimed at reducing imaging expenditures.[1–3] Professional society organizations and clinical researchers have also taken steps to better characterize the value of cardiac imaging,[4–6] while also highlighting clinical scenarios under which imaging use is particularly low-value and unlikely to improve patients’ health or management. While the Choosing Wisely campaign is perhaps the most widely recognized of these professional efforts to self-regulate use of low-value tests and procedures, it was preceded and informed, in part, by the American College of Cardiology’s (ACC) development of appropriate use criteria (AUC) for cardiac imaging stress tests.[7] These AUC have expanded to inform the use of a variety of imaging studies and invasive procedures, but cardiac stress testing has been a focal point of attention, largely due to its wide dissemination,[2] radiation risks,[8] procedural risks, expense, and association with downstream testing and procedures—some of which are invasive.[9] However, until recently, little was known about the potential long-term impact of the ACC’s appropriate use criteria on clinical decision-making in patients evaluated for ischemic heart disease.[10]

We aimed to (1) systematically review studies of cardiac stress testing appropriateness, including appropriateness within physician specialties; (2) evaluate trends over time and in response to updates of AUC; and (3) characterize leading indications for inappropriate/rarely appropriate testing.

While a recent meta-analysis provided important insights into trends in appropriateness across several cardiac imaging modalities,[10] our study differs from this prior work in important ways: we include a greater number of published studies, report a wider range of information about patients characteristics in each study, provide information about indications for inappropriate/rarely appropriate testing, perform more robust analyses of appropriateness by physician specialty (we use both meta-regression and meta-analysis to compare cardiac specialists and internists), and apply a more rigorous method for evaluating temporal trends (we pooled more studies and adjusted for AUC version). Simply stated, we add a more methodologically rigorous meta-analysis to the literature on cardiac imaging appropriateness.

## Methods

### Search Strategy

We searched PubMed (which includes the MEDLINE database and other sources) from October 1, 2005 to March 1, 2015 for English-language articles reporting stress echocardiography and radionuclide myocardial perfusion imaging (MPI) appropriateness. Our search terms included the Medical Subject Headings *exercise test*, *Cardiac Imaging Techniques*, *myocardial perfusion imaging*, *single photon emission computed tomography*, *and echocardiography*; keywords identifying cardiac imaging stress tests, including *stress test*, *thallium*, *sestamibi*, *Technetium*, *myocardial perfusion*, *MPI*, *SPECT*, and *echo*; and keywords identifying appropriateness evaluations, including approp* (for “appropriate” and variants), and inapprop* (for “inappropriate” and variants). We identified additional publications through discussion between collaborators. Our report adheres to guidelines for systematic reviews recommended by the Preferred Reporting Items for Systematic Reviews and Meta-Analyses (PRISMA) statement and Metaanalysis of Observational Studies in Epidemiology (MOOSE) group (see Supplemental materials).

### Study Selection

Two investigators (J.L. and S.B.), working independently, in duplicate, identified studies eligible for further review after screening titles or abstracts. Studies then underwent full-text retrieval and data extraction if authors reported rates of appropriate or inappropriate cardiac stress testing based on published AUC. Studies were ineligible for inclusion if they focused on special populations (e.g., transplant candidates) whose clinical characteristics made them less representative of general populations undergoing cardiac stress testing, though we did include one study that enrolled only patients with acute chest pain.[11] When multiple studies reported appropriateness outcomes on identical or overlapping populations, only studies that reported unique outcomes were included (see Table 1 footnote for more details). When a cohort was evaluated with the original and updated AUC, both cohorts were included in the meta-analysis, but in separate strata. However, for meta-regression models, only the cohort enrolled in the year closest to the publication date of the AUC was included.

**Table 1:** Characteristics of Studies Included in the Meta-analysis.

Source†	Academic medical center*	Enrollment year	No. of patients	Mean age, yr	Men, %	Diabetes, %	Dyslipidemia, %	Hypertension, %	Smoking, %	MI, %	BMI>30, %	Any revascularization, %**	CAD, %	Resting ECG normal, %	Cardiac specialist, %	Exercise stress, %	Pharmocologic stress, %
**Stress Echo 2008 AUC**																	
McCully et al, 2009	Yes	2005	298	66	52	20	66	60	54	11	NA	20	NA	41	NA	NA	NA
Mansour et al, 2010	Yes	2008	289	59	51	NA	NA	NA	NA	NA	NA	NA	NA	NA	45	49	51
Willens et al, 2013	Yes	2008	209	56	47	NA	NA	NA	NA	NA	NA	NA	NA	NA	52	NA	NA
Schmitz et al, 2013	Yes	2009–2010	300	NA	NA	NA	NA	NA	NA	NA	NA	NA	NA	NA	NA	NA	NA
Lin et al, 2013	No	2010–2011	111	51	46	11	52	44	33	NA	NA	NA	22	NA	100	NA	NA
Bhatia et al, 2013	Yes	2011	252	58	58	26	48	65	46	13	NA	8	NA	NA	50	NA	NA
**Stress Echo 2011 AUC**																	
Cortigiani et al, 2012	No	2001–2007	1552	66	56	22	45	62	21	22	NA	18	35	NA	NA	NA	100
Mansour et al, 2012	Yes	2008	289	NA	NA	NA	NA	NA	NA	NA	NA	NA	NA	NA	NA	NA	NA
Willens et al, 2013	Yes	2008	209	56	47	NA	NA	NA	NA	NA	NA	NA	NA	NA	52	NA	NA
Gertz et al, 2015	Yes	2010–2011	88	57	56	25	28	50	19	NA	NA	NA	16	NA	NA	NA	NA
Battacharyya et al, 2014	Yes	2010–2011	250	63	42	24	38	57	10	8	NA	8	NA	NA	NA	52	48
Bhatia et al, 2013	Yes	2011	252	58	58	26	48	65	46	13	NA	8	NA	NA	50	NA	NA
Willens et al, 2013	Yes	2011	209	56	47	NA	NA	NA	NA	NA	NA	NA	NA	NA	53	NA	NA
Willens et al, 2013	Yes	2011	111	58	50	NA	NA	NA	NA	NA	NA	NA	NA	NA	100	NA	NA
**Stress MPI 2005 AUC**																	
Gibbons et al, 2008	Yes	2005	284	67	63	27	78	71	48	20	41	34	NA	31	NA	NA	NA
Soine et al, 2012	Yes	2005–2008	1445	61	91	31	60	77	39	0	NA	0	0	0	NA	41	59
Soine et al, 2012	Yes	2005–2008	1377	58	48	21	42	51	18	0	NA	0	0	0	NA	63	37
Mehta et al, 2008	Yes	2006	1209	61	45	NA	NA	NA	NA	NA	NA	NA	NA	NA	NA	NA	NA
Gibbons et al, 2010	Yes	2006	284	68	67	27	78	68	54	19	39	32	NA	32	NA	NA	NA
Druz et al, 2011	Yes	2007–2008	585	64	55	NA	NA	NA	NA	NA	NA	NA	28	NA	43	57	43
Hendel et al, 2010	No	2008–2009	6351	66	59	23	73	77	12	NA	NA	36	40	NA	75	54	44
Gibbons et al, 2011	Yes	2008	273	65	67	25	77	73	52	21	44	33	NA	34	NA	NA	NA
Gupta et al, 2011	Yes	2008–2009	314	62	52	24	56	64	25	12	NA	8	33	NA	38	NA	NA
Oliveira et al, 2014	No	2008–2009	367	65	64	27	50	61	17	NA	20	NA	NA	NA	NA	NA	NA
Gholamrezanezhad et al, 2011	No	2009	291	55	43	22	60	50	15	11	NA	14	NA	NA	NA	NA	NA
**Stress MPI 2009 AUC**																	
Carryer et al, 2010	Yes	2005	281	67	63	27	78	71	48	20	41	34	NA	31	NA	NA	NA
Aldweib et al, 2013	Yes	2006	1199	64	57	28	73	82	58	21	45	NA	NA	NA	NA	65	35
Doukky et al, 2013	No	2007–2010	1511	59	57	22	46	56	12	2	NA	6	18	91	8	NA	NA
Oliveira et al, 2014	No	2008–2009	367	65	64	27	50	61	17	NA	20	NA	NA	NA	NA	NA	NA
Gholamrezanezhad et al, 2011	No	2009	291	55	43	22	60	50	15	11	NA	14	NA	NA	NA	NA	NA
Nelson et al, 2012	Yes	2009	150	61	99	35	77	87	43	38	NA	21	NA	NA	NA	NA	NA
Nelson et al, 2012	Yes	2009	150	65	57	21	62	74	23	29	NA	23	NA	NA	47	NA	NA
Koh et al, 2011	Yes	2009	1623	61	59	31	75	71	14	NA	NA	NA	NA	NA	93	59	41
Lalude et al, 2014	Yes	2009	420	56	44	NA	NA	NA	NA	NA	NA	NA	NA	NA	NA	NA	NA
Bohossian et al, 2015	Yes	2010–2011	133	NA	NA	NA	NA	NA	NA	NA	NA	NA	NA	NA	NA	100	NA
Saifi et al, 2013	No	2010–2011	11845	NA	NA	NA	NA	NA	NA	NA	NA	NA	NA	NA	NA	NA	NA
Lin et al, 2013	No	2010–2011	338	57	66	25	76	76	38	NA	NA	NA	49	NA	100	NA	NA
Gertz et al, 2015	Yes	2010–2011	369	62	55	31	62	83	29	NA	NA	NA	34	NA	NA	NA	NA
Moralidis et al, 2013	Yes	2010–2011	3032	66	59	31	66	80	19	NA	NA	24	44	NA	92	29	71
Johnson et al, 2014	No	2010	205	NA	NA	NA	NA	NA	NA	NA	NA	NA	NA	NA	100	NA	NA
Winchester et al, 2014	Yes	2010–2011	582	NA	96	41	76	82	26	41	NA	36	41	NA	38	NA	NA
Bohossian et al, 2015	Yes	2011–2013	212	NA	NA	NA	NA	NA	NA	NA	NA	NA	NA	NA	NA	100	NA
Singh et al, 2014	No	2011	328	67	56	33	65	80	14	NA	NA	NA	NA	NA	50	NA	NA
Johnson et al, 2014	No	2012	206	NA	NA	NA	NA	NA	NA	NA	NA	NA	NA	NA	100	NA	NA
Medolago et al, 2014	No	2013	2134	67	67	NA	NA	NA	NA	NA	NA	NA	49	NA	98	62	38
Mahajan et al, 2015	Yes	2013–2014	403	62	48	31	67	71	27	NA	50	18	27	NA	32	NA	NA

Abbreviations: AUC, Appropriate use criteria; Echo, echocardiography; MPI, myocardial perfusion imaging; NA, not available; PTCA, percutaneous transluminal coronary angioplasty; CABG, coronary artery bypass grafting

*Defined as studies whose patients were stress tested only at academic medical centers.

**Reflects prevalence of PTCA and may not include CABG for Bhatia et al, 2013; Cortigiani et al, 2012; Battacharyya et al, 2014; Hendel et al, 2010; Doukky et al, 2013; Nelson et al, 2012; and Moralidis et al, 2013.

^†^For Mehta et al, 2008, authors did not report demographic data for patients who were unclassified by AUC.

Note about cohort overlap: There are several studies or cohorts that appear to have significant overlap but do not (though some overlap cannot be ruled out). For example, Gibbons et al, 2008, Gibbons et al, 2010, and Gibbons et al, 2011 (all under "Stress MPI 2005 AUC") enrolled cohorts in different years (note that the 2008 and 2010 studies have the same sample size but the enrollment years and demographic characteristics of the cohorts differ; they are therefore not identical cohorts). Similarly, Soine et al, 2012 ("Stress MPI 2005 AUC") enrolled two large cohorts over the same period of years but one cohort was enrolled from University of Washington Medical Center (n = 1377) and the second cohort was enrolled from the Veterans Health Administration of Puget Sound (n = 1445); the two cohorts are therefore not identical. Willens et al, 2013 ("Stress Echo 2008 AUC" and "Stress Echo 2011 AUC") used a complex study design and evaluated one cohort with both 2008 and 2011 Stress Echo AUC (n = 209, enrollment year = 2008) but analyses of these cohorts were not pooled so as to avoid double-counting. Willens et al, 2013 also enrolled two additional cohorts (n = 209 and n = 111; enrollment year = 2011 for both); though one of these cohorts has the same sample size as the cohort enrolled in 2008, it is not an identical cohort. Nelson et al, 2012 ("Stress MPI 2009 AUC") enrolled two cohorts of 150 patients in 2009 but one cohort was enrolled from the Miami VA Medical Center and the other was enrolled from the University of Miami Medical Group. Johnson et al, 2014 ("Stress MPI 2009 AUC") enrolled similarly sized cohorts but one cohort was enrolled in 2010 (n = 205) and the other was enrolled in 2012 (n = 206).

### Data Extraction

Using a standardized protocol and reporting form, data were extracted on the following characteristics: (1) identifying information (first author, journal, country, institution, publication year); (2) AUC used (stress echocardiography 2008 or 2011 AUC, stress MPI 2005 or 2009 AUC); (3) patient characteristics (mean age, percentage of male patients, percentage of patients with a history of diabetes, hypertension, hyperlipidemia, body mass index>30, myocardial infarction (MI), percutaneous transluminal coronary angioplasty (PTCA), or coronary artery bypass grafting (CABG); (4) stress test characteristics (test used, type of stressor); (5) appropriateness patterns, including appropriateness stratified by physician specialty; and (6) indications for inappropriate/rarely appropriate testing. We recalculated appropriateness rates when authors excluded patients whose studies were unclassified, but did not include papers that did not report the number of patients who were unclassified. Disagreements between reviewers were resolved through discussion.

### Statistical Analysis

The primary outcomes were the proportions of appropriate, inappropriate/rarely appropriate, uncertain/may be appropriate, and unclassified cardiac imaging stress tests. Patient characteristics were summarized after weighting by each study’s sample size. When studies reported that no patients were categorized as unclassified, a 0.5 correction factor was added to that outcome to facilitate calculation of a rate and standard error. Appropriateness estimates were pooled using the DerSimonian—Laird random-effects model to account for between-study heterogeneity attributable to differences in patient populations and clinician practice patterns. Statistical heterogeneity was also assessed with the Cochran Q statistic (a weighted sum of squared differences between studies with a χ2 distribution) and *I*^2^ statistic, which is derived from the Q statistic ([Q − df/Q] x 100) and estimates the proportion of overall variation attributable to between-study heterogeneity rather than chance. Because rates of uncertain/may be appropriate and unclassified patients tended to be low, we log transformed these values to more accurately estimate their standard errors and confidence intervals. To assess for publication bias, we constructed funnel plots (standard error versus appropriateness rates) stratified by AUC and performed the Egger test when at least 10 studies were present. None of these plots or statistical tests raised concerns for publication bias.

### Meta-regression for temporal trends and effects of AUC updates

We performed meta-regression to assess temporal trends in appropriate and inappropriate/rarely appropriate cardiac stress testing. Meta-regression in this context is limited by the possibility of ecological bias (sometimes referred to as “aggregation bias” or “ecological confounding”),[12] since appropriateness rates in different cohorts over time may not reflect overall trends in appropriateness. We hypothesized that academic setting, prevalence of risk factors for ischemic heart disease (gender, age, comorbidities), and physician specialty would influence rates of appropriateness. However, because many studies reported only a few risk factors, we limited our patient covariates to gender and age, so as not to significantly reduce sample size for these regression models.[12] Separate models pooled all stress echocardiography or MPI studies, and we included an indicator for the specific AUC used. The key variable in these models was time, as captured by the midpoint of the enrollment period. To avoid double-counting, when the same stress echocardiography or MPI cohort was evaluated with original and updated AUC, we used the AUC whose publication date was closest to the enrollment dates.

We also used meta-regression to examine whether updated stress echocardiography and MPI AUC were associated with a reduction in unclassified patients, and to test whether cardiac specialists (cardiologists and cardiac surgeons) and internists had different rates of appropriate and inappropriate cardiac stress testing. Most studies reporting specialty appropriateness categorized physicians as cardiac specialists or non-cardiac specialists, but we considered the latter to be internists based on national referral patterns.[1] These regression models included indicators for the AUC version and presence of cardiac specialists (physician specialty model). We also explored performing a comparison of the pre-2005 period to the post-2005 period but were unable to do so because patient enrollment for all studies included in our meta-analysis began during or after 2005, with the exception of Cortigiani et al 2012. A 2-tailed P-value of <0.05 was considered statistically significant. Analyses were performed in Stata (version 14, StataCorp, College Station, Texas) with the metan family of functions.

## Results

### Literature Search

**Fig 1:**
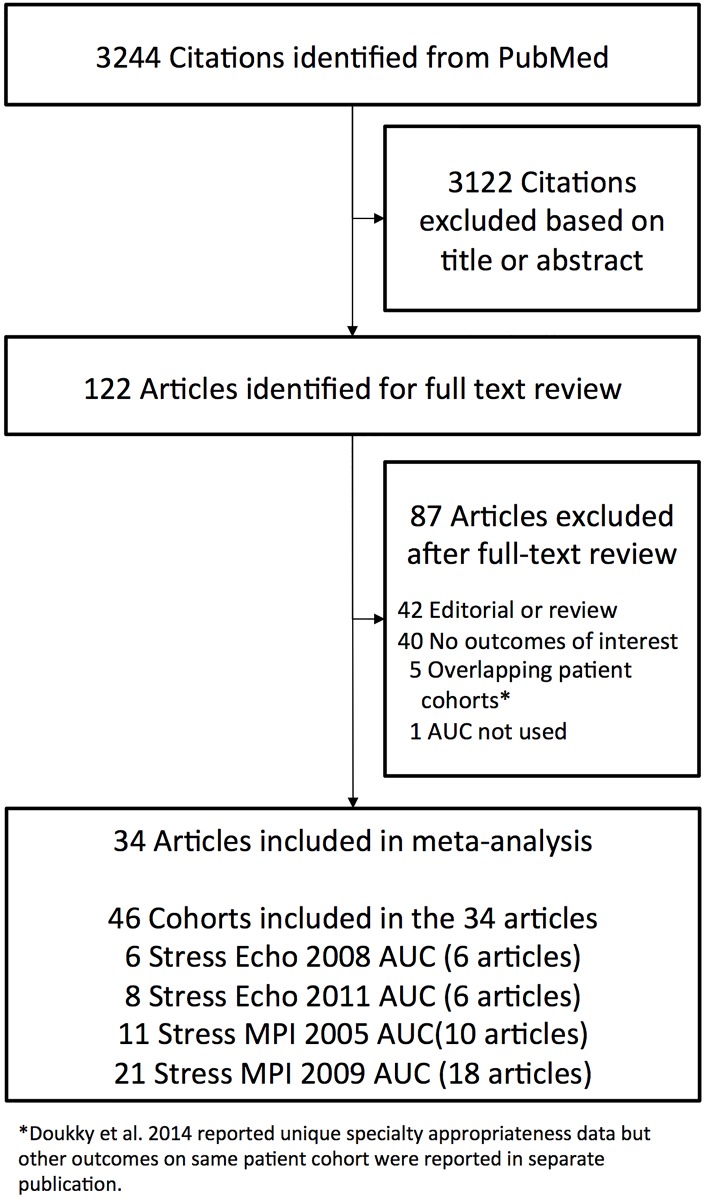
Flow Diagram of the Literature Search and Study Selection.

Our literature search yielded a total of 3,244 citations, of which 3,122 were excluded after initial screening of abstracts or titles (Fig 1). Of the remaining 122 citations, 34 met inclusion criteria and were selected for full-text review and data extraction. These articles included 6 articles and 6 cohorts for the stress echocardiography 2008 AUC,[11, 13–17] 6 articles and 8 cohorts for the stress echocardiography 2011 AUC,[13, 17–21] 10 articles and 11 cohorts for the stress MPI 2005 AUC,[22–31] and 18 articles and 21 cohorts for the stress MPI 2009 AUC.[14, 20, 23, 30, 32–45] Some studies contributed multiple cohorts to our meta-analysis. For example, for stress echocardiography 2011 AUC, Willens et al contributed three cohorts (three separate cohorts that underwent testing in August-September 2008, July-September 2011, and October-December 2011).[17] Similarly, for stress MPI 2005 AUC, Soine et al contributed two cohorts (one cohort underwent testing at University of Washington Medical Center and the second cohort underwent testing at the Veterans Health Administration of Puget Sound).[31] Each cohort is separately presented in the figures (note that enrollment dates are rounded to the nearest year).

The characteristics of these studies and their 41,578 participants are shown in Table 1. The mean age was 63.3 years, 40.4% were women, 12.7% had a prior history of myocardial infarction (reported in 16 studies), and 22.3% had a prior history of revascularization (reported in 18 studies). Overall, population characteristics were generally similar across studies.

### Rates of appropriate and inappropriate testing

**Fig 2:**
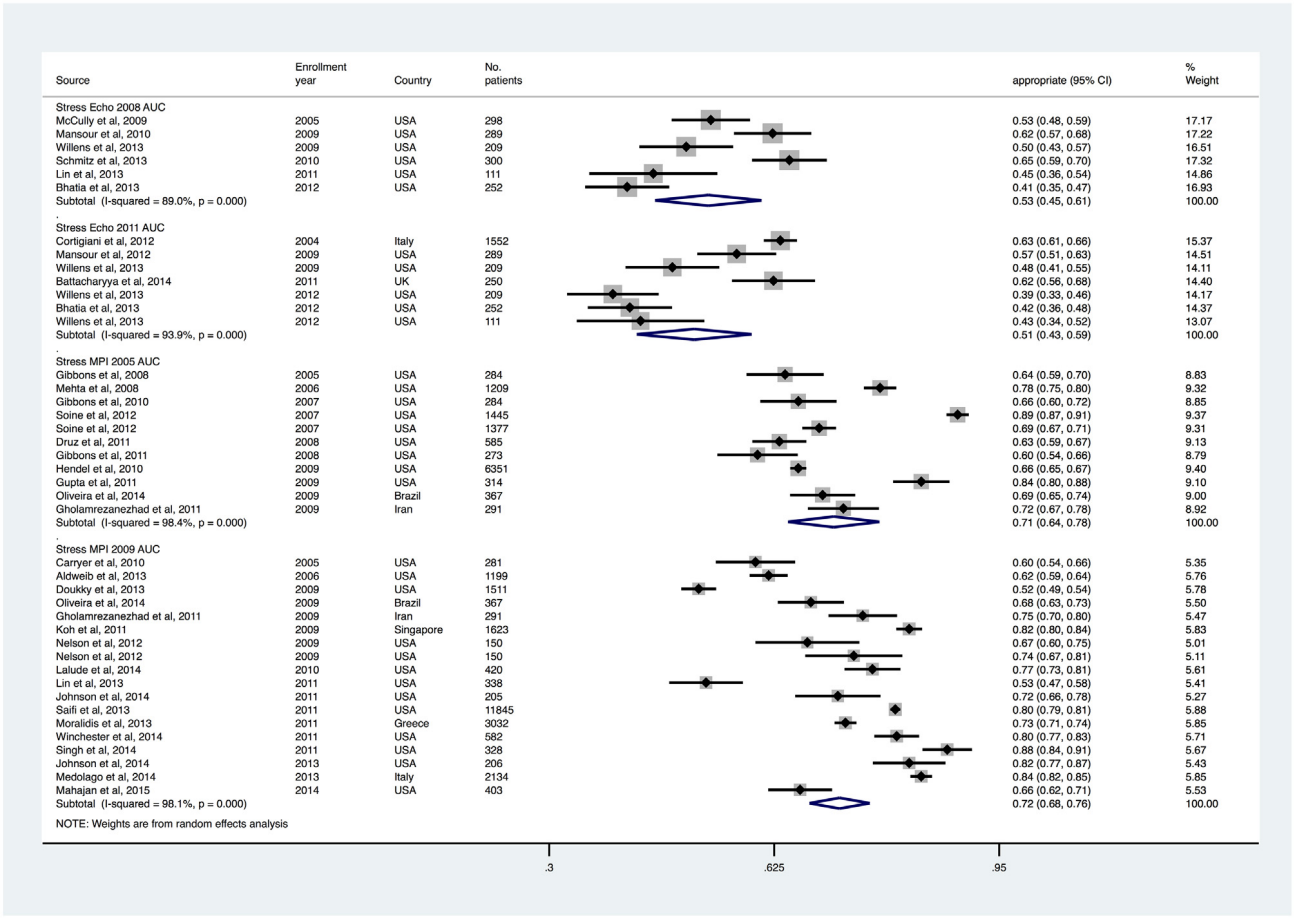
Appropriate Use Rates of Stress Echocardiography and MPI, Sorted by Patient Enrollment Year.

**Fig 3:**
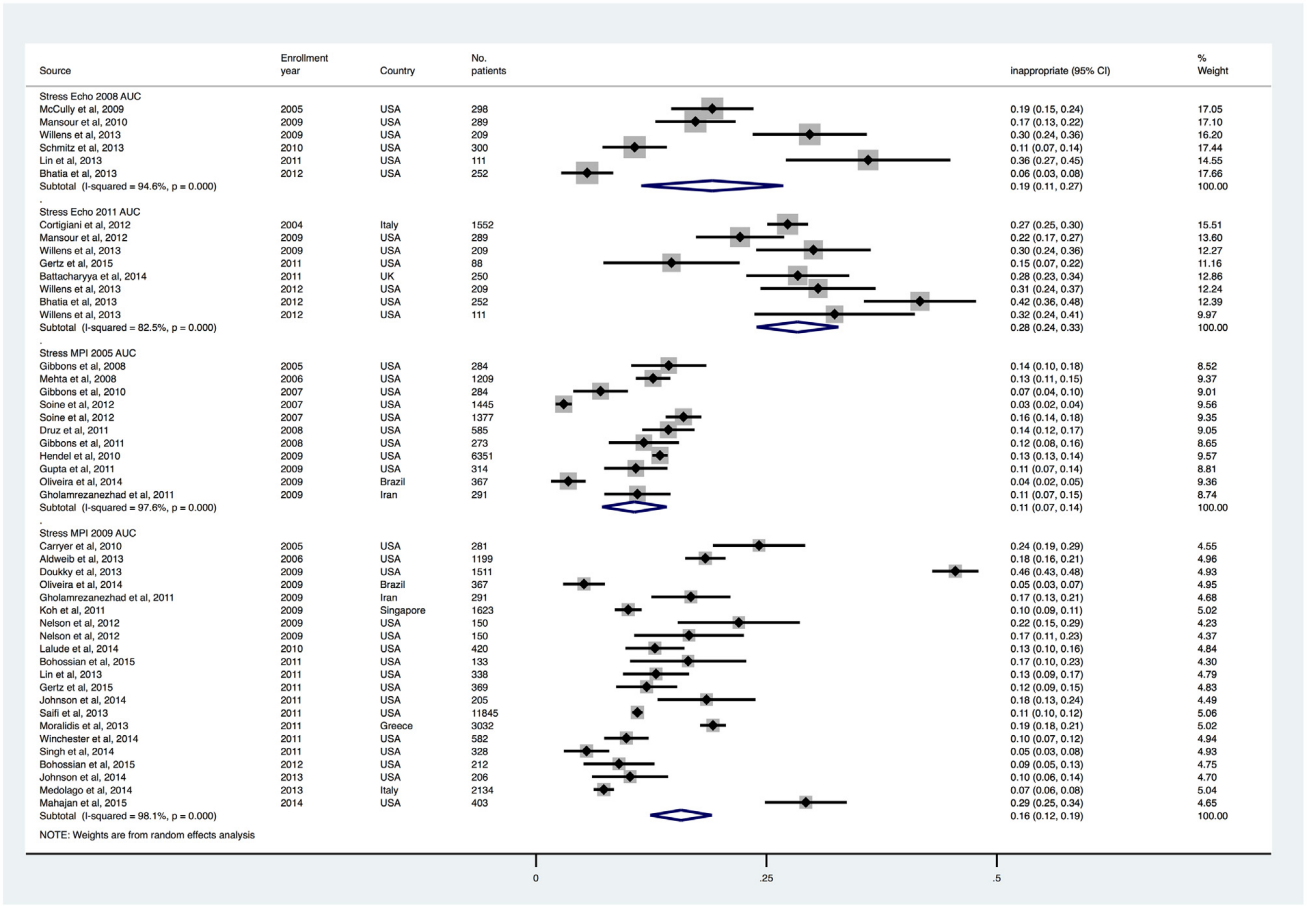
Inappropriate/Rarely Appropriate Use Rates of Stress Echocardiography and MPI, Sorted by Patient Enrollment Year.

**Fig 4:**
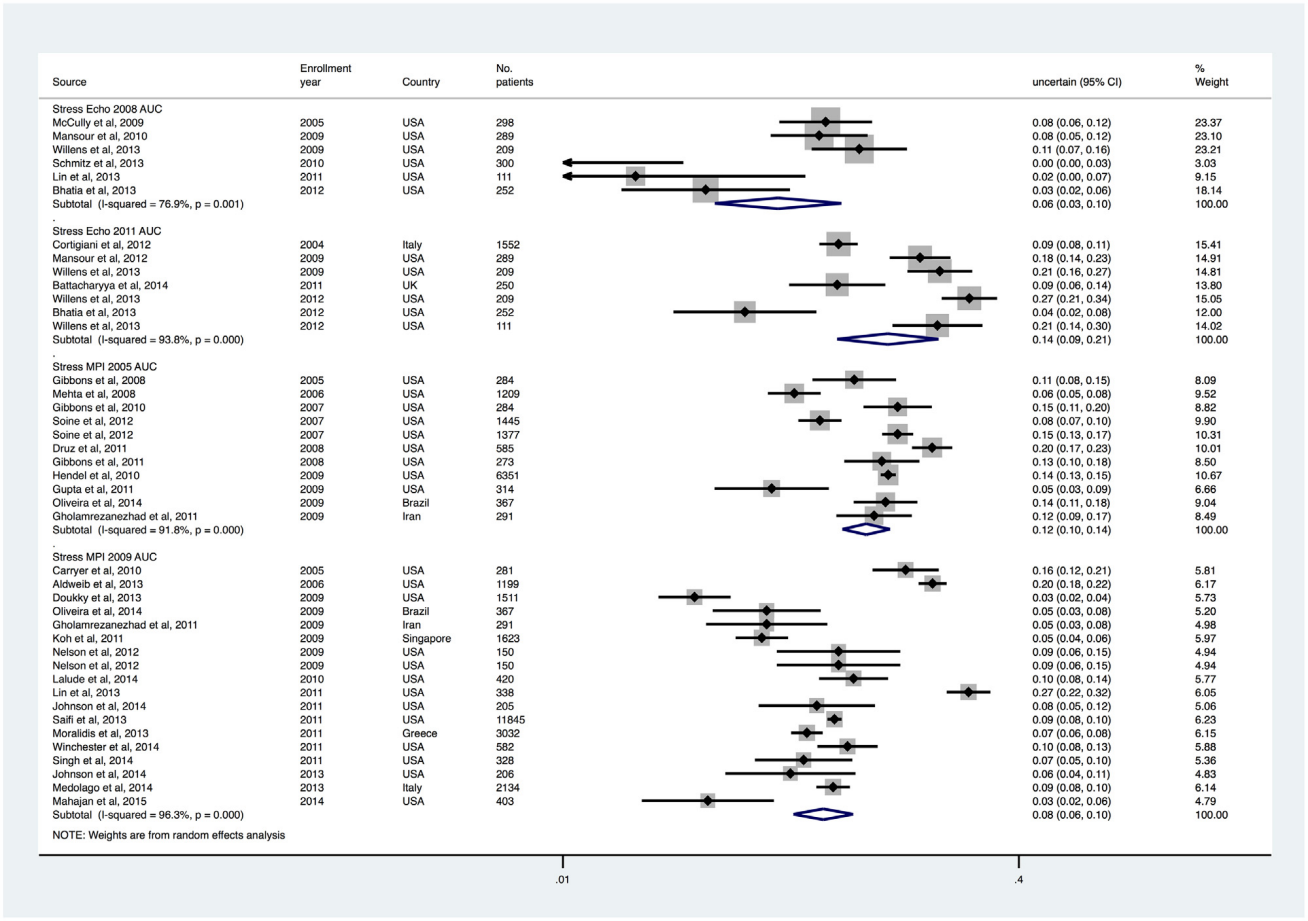
Uncertain/May be Appropriate Use Rates of Stress Echocardiography and MPI, Sorted by Patient Enrollment Year.

**Fig 5:**
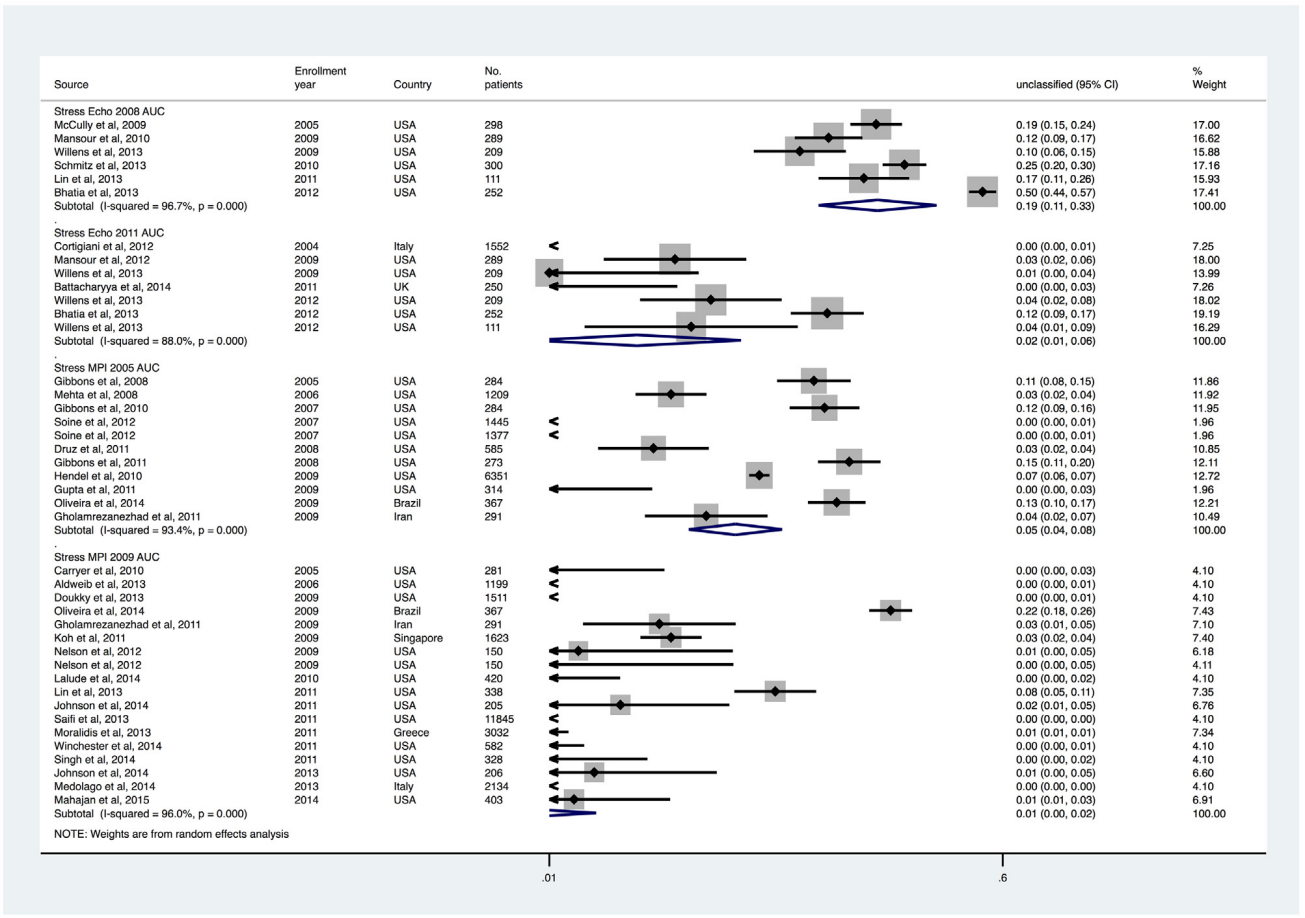
Unclassified Use Rates of Stress Echocardiography and MPI, Sorted by Patient Enrollment Year.

Appropriate cardiac stress testing rates were 53.0% (95% CI, 45.3% to 60.7%) and 50.9% (95% CI, 42.6% to 59.2%) with stress echocardiography 2008 and 2011 AUC, and 71.1% (95% CI, 64.5% to 77.7%) and 72.0% (95% CI, 67.6% to 76.3%) with stress MPI 2005 and 2009 AUC, respectively (Figs 2 and 3). Inappropriate/rarely appropriate cardiac stress testing rates were 19.1% (95% CI, 11.4% to 26.8%) and 28.4% (95% CI, 23.9% to 32.8%) with stress echocardiography 2008 and 2011 AUC, and 10.7% (95% CI, 7.2% to 14.2%), and 15.7% (95% CI, 12.4% to 19.1%) with stress MPI 2005 and 2009 AUC (Figs 4 and 5).

### Temporal trends in appropriate and inappropriate/rarely appropriate testing

We examined temporal trends and separately pooled all stress echocardiography or MPI studies, while controlling for AUC version, academic setting, population age, and population gender. For stress echocardiography and MPI, the average annual changes in appropriate testing were -1.9% (95% CI, -4.6% to 0.8%; adjusted change = +1.1%; 95% CI, -11.4% to 13.7%) and +1.9% (95% CI, -0.6% to 4.4%; adjusted change = +1.7%; 95% CI, -1.5% to 4.9%), and the average annual changes in inappropriate/rarely appropriate testing were +0.9% (95% CI, -1.7% to 3.5%; adjusted change = +2.8%; 95% CI, -8.5% to 14.1%) and -0.9% (95% CI, -2.8% to 1.1%; adjusted change = -0.2%; 95% CI, -2.8% to 2.4%), respectively.

In a sensitivity analysis, we attempted to analyze trends in appropriateness within the same institution, but these meta-regression models were not estimable due to limited sample size. However, we provide raw appropriateness rates from these studies: for stress echocardiography, one study from University of Miami Miller School of Medicine with three cohorts reported appropriate rates of 49.8% (2008 AUC, patient recruitment year 2009), 39.2% (2011 AUC, patient recruitment year 2012), and 43.2% (2011 AUC, patient recruitment year 2012). For stress MPI, three studies from Mayo Clinic reported appropriate rates of 64.1% (2005 AUC, patient recruitment year 2005), 66.0% (2005 AUC, patient recruitment year 2007), and 60.1% (2005 AUC, patient recruitment year 2008).[24–26]

### Uncertain/may be appropriate tests and changes in unclassified tests after AUC updates

Rates of uncertain/may be appropriate and unclassified stress tests for both modalities were generally low, but tended to be higher for stress echocardiography compared to stress MPI. The proportion of testing that was considered uncertain/may be appropriate was 5.7% (95% CI, 3.4% to 9.5%) and 13.9% (95% CI, 9.2% to 20.3%) with stress echocardiography 2008 and 2011 AUC, and 11.6% (95% CI, 9.6% to 14.1%) and 8.2% (95% CI, 6.5% to 10.5%) with stress MPI 2005 and 2009 AUC, respectively. The proportion of testing that was unclassified was 19.4 (95% CI, 11.4% to 33.0%) and 2.2% (95% CI, 0.9% to 5.6%) with stress echocardiography 2008 and 2011 AUC, and 5.4% (95% CI, 3.5% to 8.2%) and 0.7% (95% CI, 0.3% to 1.5%) with stress MPI 2005 and 2009 AUC, respectively.

A test for differences in unclassified rates demonstrated that the updated stress echocardiography AUC in 2011 was associated with a significant reduction in the proportion of these tests (relative reduction = 79%, p = 0.04). There was no evidence of a reduction in unclassified studies after the updated stress MPI criteria were released in 2009 (relative reduction = 64%, p = 0.25).

### Appropriateness by physician specialty

**Fig 6:**
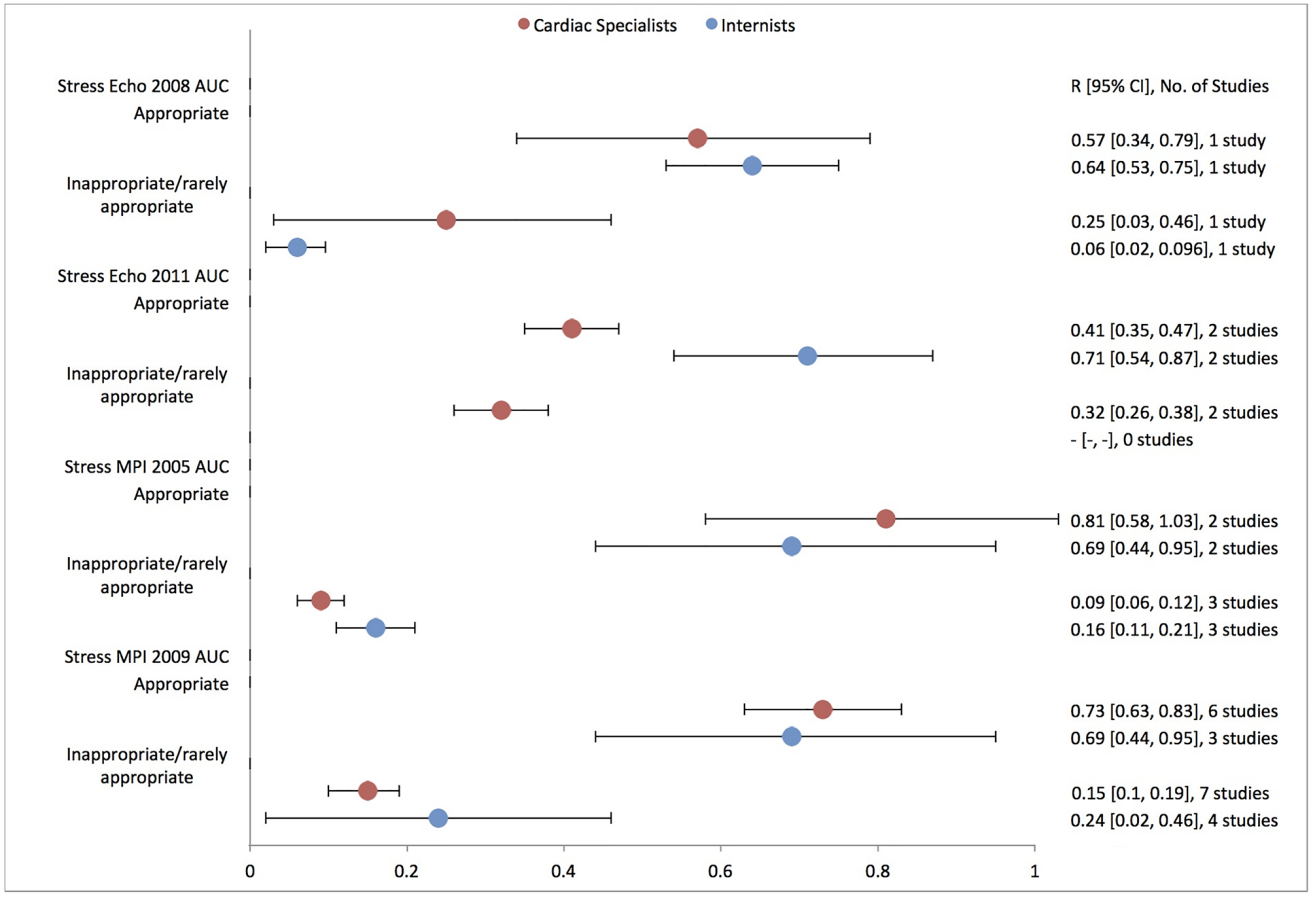
Physician Specialty Appropriate and Inappropriate Use Rate.

Only 11 studies reported appropriateness rates by physician specialty,[14, 15, 17, 22, 27, 36, 39, 40, 44–46] and 3 of these studies focused solely on cardiologists.[14, 17, 36] Pooled appropriateness rates from these specialty studies are reported in Fig 6. A test for heterogeneity demonstrated no significant difference in the proportion of appropriate stress echocardiograms or MPIs ordered by cardiac specialists compared to internists (stress echocardiogram difference = -7.3% [95% CI, -70.7% to 56.2%]; stress MPI difference = +7.5% [95% CI, -9.4% to 24.4%]; both with internists as the reference group), and no significant difference in the proportion of inappropriate/rarely appropriate stress echocardiograms or MPIs (stress echocardiogram difference = +12.5% [95% CI, -45.8% to 70.8%]; stress MPI difference = -10.5% [95% CI, -23.8% to 2.8%]).

### Indications for inappropriate testing

Indications for inappropriate/rarely appropriate cardiac stress testing (Table 2) were reported by 7 stress echocardiography studies[13–17, 19, 20] and 20 stress MPI studies.[14, 20, 22, 25, 26, 28–30, 32, 34–42, 44, 45] The three most frequent indications for inappropriate/rarely appropriate testing tended to be preoperative evaluation (range 0.0% to 90.0% for stress echocardiography, 0.0% to 75.0% for stress MPI); evaluation of symptomatic patients (often because they were low risk), had an interpretable electrocardiogram, and could exercise (range 10.5% to 44.4% for stress echocardiography, 5.1% to 57.0% for stress MPI); and evaluation of asymptomatic patients (range 4.0% to 65.0% for stress echocardiography, 0.0% to 60.0% for stress MPI).

**Table 2:** Indications for Inappropriate/Rarely Appropriate Cardiac Stress Test Use.

Source	All (>95%) major inappropriate/rarely appropriate indications reported	Preoperative evaluation, %	Followup of prior testing, %	Post revascularization, %	Symptomatic patient, %	Asymptomatic patient, %	Other, %*†
**Stress Echo 2008 AUC**							
McCully et al, 2009	Yes	18	2	16	11	54	0
Mansour et al, 2010	Yes	40	NA	NA	44	12	NA
Lin et al, 2013	No	NA	NA	5	23	65	NA
**Stress Echo 2011 AUC**							
Cortigiani et al, 2012	Yes	4	7	32	33	21	3
Willens et al, 2013	No	16	6	NA	44	8	NA
Gertz et al, 2015	Yes	85	0	0	8	8	0
Bhatia et al, 2013	No	90	NA	NA	NA	NA	NA
Willens et al, 2013	No	8	17	NA	34	5	NA
Willens et al, 2013	No	NA	36	NA	44	6	NA
**Stress MPI 2005 AUC**							
Gibbons et al, 2008	Yes	15	12	12	12	49	0
Mehta et al, 2008	No	28	NA	NA	57	6	NA
Gibbons et al, 2010	Yes	2	19	32	28	17	0
Druz et al, 2011	Yes	7	0	0	43	50	0
Hendel et al, 2010	No	4	4	24	16	45	NA
Oliveira et al, 2014	Yes	23	0	39	31	8	0
**Stress MPI 2009 AUC**							
Carryer et al, 2010	No	10	28	10	NA	43	NA
Aldweib et al, 2013	Yes	33	31	17	5	10	NA
Doukky et al, 2013	No	3	NA	NA	34	28	NA
Oliveira et al, 2014	No	11	NA	21	16	NA	21
Nelson et al, 2012	No	32	20	NA	16	16	NA
Nelson et al, 2012	Yes	30	6	NA	NA	60	NA
Koh et al, 2011	Yes	59	3	2	21	11	NA
Lalude et al, 2014	Yes	4	4	2	46	41	NA
Lin et al, 2013	No	NA	NA	11	18	48	NA
Gertz et al, 2015	Yes	77	0	2	9	11	0
Moralidis et al, 2013	No	19	11	39	10	21	0
Johnson et al, 2014	Yes	39	0	17	33	0	11
Winchester et al, 2014	Yes	12	12	12	18	44	0
Singh et al, 2014	No	44	NA	NA	NA	NA	NA
Medolago et al, 2014	Yes	NA	14	24	29	28	NA
Mahajan et al, 2015	No	20	12	NA	52	NA	NA

Abbreviations: AUC, Appropriate use criteria; Echo, echocardiography; MPI, myocardial perfusion imaging; NA, not available

*Imputed as 0 if sum of other categories exceeded 98%, a threshold selected to account for rounding errors in author reporting

^†^Reflects authors' report unless imputed as 0 or not reported.

## Discussion

By systematically reviewing studies of cardiac stress testing AUC, we found that rates of appropriate use tended to be lower for stress echocardiography compared to stress MPI, and that rates of inappropriate/rarely appropriate use tended to be higher. In the patient recruitment years of 2005 to 2014, we also found that rates of appropriate testing did not change significantly for stress echocardiography or MPI. Importantly, we showed that rates of unclassified stress echocardiograms fell after release of the 2011 AUC, whereas no significant changes were identified after updated stress MPI AUC were released. We did not find differences in appropriateness between physician specialties, though these analyses were substantially limited by sparse reporting. Finally, we found significant variability in indications for inappropriate/rarely appropriate cardiac stress tests, with preoperative testing and testing of low-risk symptomatic or asymptomatic patients representing leading indications.

Our study demonstrates that early efforts of the ACC’s Appropriateness Criteria Working Group have had durable and far-reaching consequences on the trajectory of academic inquiry into appropriate testing, with more than 41 diverse cohorts evaluated since publication of the original 2005 AUC. These evaluations have also extended into the community setting, though academic medical centers remain the dominant site for AUC evaluation. While the rapid growth in cardiac imaging that spurred initial efforts to develop AUC may be slowing—at least for stress MPI—the total number of cardiac stress test referrals in US ambulatory settings has not changed in recent years, and expenditures on inappropriate tests remain substantial.[1]

The main findings of our study are similar to those from a recently published meta-analysis[10] of cardiac imaging appropriateness, but there are important methodological differences: Fonseca et al analyzed 10 stress MPI 2009 articles with 11 cohorts whereas we analyzed 18 stress MPI 2009 with 21 cohorts; we present clinical characteristics from study cohorts that were not presented in Fonseca et al’s work, including the prevalence of diabetes, dyslipidemia, hypertension, smoking, coronary artery disease/myocardial infarction, and obesity; provide information about indications for inappropriate/rarely appropriate testing, which was absent in Fonseca et al; perform more robust analyses of appropriateness by physician specialty (we use both meta-regression and meta-analysis to compare cardiac specialists and internists); and apply a more rigorous method for evaluating temporal trends (we pooled more studies and adjusted for AUC version, whereas Fonseca et al estimated separate models [and therefore had smaller sample sizes] for each AUC version). In the context of AUC design, our study suggests that the potential effects of AUC are unclear, but these analyses are limited by the absence of a control group, and they are vulnerable to ecological bias.[12] We found no conclusive evidence of a trend over time in appropriate or inappropriate/rarely appropriate stress echocardiograms or MPIs. These results are in agreement with the work of Fonseca et al,[10] though our study samples differed (we captured more recently published studies), we used enrollment year instead of publication year as our measure of time, and we included an indicator in our meta-regression models for the AUC version used rather than separately treating studies that used different AUC. Our findings are also similar to the results of another recent meta-analysis that focused on stress MPI.[47] It is important to note, however, that there is substantial uncertainty about the extent to which findings within different cohorts in our meta-analysis reflect general trends.[12] Further, we did not account for geographic variation in appropriate and inappropriate use of cardiac imaging, which may be an important source of confounding.

Notably, a greater number of stress MPI publications reported the results of quality improvement initiatives, such as one study that reported the effects of FOCUS (Formation of Optimal Cardiovascular Utilization Strategies), a Web-based community and quality improvement tool.[43] We hypothesized that higher expenditures on stress MPI and expansion of administrative controls such as prior authorization requirements, in combination with widening public concerns about radiation exposure, may have engendered a climate of urgency in the context of stress MPI. It is also possible that these factors may have had the unintended consequence of causing a shift in ordering practices to stress echocardiography, in order to avoid stress MPI in questionable scenarios or other scenarios. Nonetheless, more concerted efforts to increase appropriate use of stress echocardiography and MPI and reduce inappropriate/rarely appropriate use are needed.

Our findings have important implications for insurers and policymakers. A substantial proportion of cardiac imaging stress tests remain inappropriate/rarely appropriate, and our pooled estimates—based largely on studies from academic medical centers—may underestimate the inappropriate/rarely appropriate use of this technology and overestimate its appropriate use in the community. Notably, some studies, such as Doukky et al, focused on patients undergoing testing in a community setting.[35] These inappropriate/rarely appropriate tests increase healthcare expenditures and are less likely to yield positive findings or improve patients’ health outcomes. It is important to recognize that a goal of zero inappropriate/rarely appropriate use is not only unrealistic but undesirable, as each patient represents unique considerations. While the optimal proportion is unknown, it is likely in the range of 10%, though no formal benchmarks have been proposed. Related to this, the small proportion of unclassified studies and relatively modest proportion of studies with uncertain appropriateness suggest that AUC may be an effective tool for evaluating the value of cardiac imaging stress tests, independent of prior authorization mechanisms and radiology benefits managers. Thus, wider incorporation and application of AUC, particularly in integrated health systems and accountable care organizations, could reduce the need for these alternate methods for constraining unnecessary utilization.

Introduction of the 2013 multimodality AUC adds calcium scoring and nonimaging exercise testing to the cohort of technologies subject to appropriate use review. We attempted to integrate the multimodality AUC into our meta-analysis but no studies rigorously implementing it were available at the time of our literature search. However, we did adopt the terminology of the multimodality AUC (e.g., “rarely appropriate” instead of “inappropriate”) to more closely align our results with current interpretation of appropriateness. Assessing its effects, particularly in cohorts that have previously been evaluated with earlier AUC versions, will provide important insights into the overall effect of multimodality criteria, with possible implications for insurers and policymakers. In the Prospective Multicenter Imaging Study for Evaluation of Chest Pain (PROMISE) trial,[6] all patients had chest pain, shortness of breath, or other symptoms as well as cardiovascular risk factors, and therefore would be considered appropriate candidates for cardiac imaging stress tests by these criteria. However, the routine performance of cardiac stress testing in patients without symptoms remains an important issue.[1]

Our study has several limitations. The majority of AUC evaluations were set in academic medical centers, where clinicians often care for higher-risk patients, may be more aware of AUC, and typically face weaker financial incentives to perform cardiac imaging stress tests. Because of small sample size and sparse reporting, we were unable to include a robust set of covariates in our examination of temporal trends. Moreover, meta-regression has significant limitations, including ecological bias (sometimes referred to as “aggregation bias” or “ecological confounding”),[12] and confounding from omitted variables (such as geographic variation in appropriate and inappropriate use of cardiac imaging, and clinical differences in the patient populations referred for stress echocardiography versus MPI), a risk shared by other analytic models of non-randomized, observational data. Further, the absence of a control group in our study attenuated our ability to causally link temporal changes in appropriateness to AUC development. Other ecological factors, including diffusion of radiology benefit managers and prior authorization programs, reductions in Medicare reimbursement, and the Choosing Wisely campaign, may also have contributed. In addition, application of AUC was not standardized across studies, so use of different methodologies could lead to different conclusions about appropriateness.

Recent AUC versions perform well for definitively categorizing the vast majority of stress echocardiograms and MPIs, but we found no conclusive evidence that diffusion of AUC increased the appropriate use of stress echocardiography or MPI. Overall rates of inappropriate/rarely appropriate testing are relatively low in academic settings, and integration of appropriateness guidelines in both academic and community settings may be an effective approach to further optimizing the inappropriate/rarely appropriate use of cardiac stress testing and its associated costs and patient harms.

## Supporting Information

S1 Data Data File for Meta-analysis. (CSV)

S1 Text PRISMA Checklist. (DOCX)

S2 Text MOOSE Checklist. (DOC)

## Abbreviations

ACC: American College of Cardiology
AUC: Appropriate use criteria
MPI: myocardial perfusion imaging
CABG: coronary artery bypass grafting
MI: myocardial infarction
MOOSE: Metaanalysis of Observational Studies in Epidemiology
PRISMA: Preferred Reporting Items for Systematic Reviews and Meta-Analyses
PROMISE: Prospective Multicenter Imaging Study for Evaluation of Chest Pain
PTCA: percutaneous transluminal coronary angioplasty
