# Supplementary material for: Appropriate Use of Cardiac Stress Testing with Imaging: A Systematic Review and Meta-Analysis
Source: PLoS One. 2016 Aug 18;11(8):e0161153. doi: 10.1371/journal.pone.0161153 (PMC4990235; doi:10.1371/journal.pone.0161153)
Supplement: S2 Text — (DOC) [file pone.0161153.s003.doc]

**MOOSE Checklist**

Title: Appropriate Use of Cardiac Stress Testing with Imaging: A Systematic Review And Meta-analysis

Authors:

Joseph A. Ladapo, MD, PhD^1^, Saul Blecker, MD, MHS^1^, Michael O'Donnell^2^, Saahil A. Jumkhawala^3^, and Pamela S. Douglas, MD^4^

^1^Departments of Population Health and Medicine, New York University School of Medicine, New York, NY; ^2^New York University School of Medicine, New York, NY; ^3^New York University, New York, NY; ^4^Department of Medicine, Duke University School of Medicine, Durham, NC

Address for Correspondence

Joseph A. Ladapo, MD, PhD

New York University School of Medicine

Department of Population Health

550 First Avenue, VZ30 6th Fl, 614

New York, NY 10016

Phone: 646-501-2561

Fax: 212-263-4983

Email: joseph.ladapo@nyumc.org

| **Criteria** | | **Brief description of how the criteria were handled in the meta-analysis** |
| --- | --- | --- |
| **Reporting of background should include** | |  |
| √ | Problem definition | Cardiac imaging has advanced physicians’ ability to diagnose and treat a variety of diseases, but rapid growth in the utilization and cost of imaging technology has spurred public and private insurers to scrutinize its use and construct policies aimed at reducing imaging expenditures. Appropriate use criteria (AUC) for cardiac imaging stress tests address concerns about utilization growth, high costs, and radiation safety. To address limitations of prior studies of cardiac imaging stress test appropriateness, we systematically reviewed appropriateness, including appropriateness within physician specialties, evaluated trends over time and in response to updates of AUC, and characterized leading indications for inappropriate/rarely appropriate testing. |
| √ | Hypothesis statement | Appropriateness differs by study modality (stress echocardiography vs. radionuclide myocardial perfusion imaging [MPI]), across specialties, and over time. |
| √ | Description of study outcomes | Appropriateness rates of cardiac stress testing and inappropriate indication rates. |
| √ | Type of exposure or intervention used | Stress echocardiography or MPI |
| √ | Type of study designs used | All designs were eligible, though only observational studies were found using the search and inclusion criteria |
| √ | Study population | Populations in which stress echocardiography or MPI imaging were performed to detect or evaluate coronary artery disease; excluded special populations such as transplant candidates or patients undergoing cardiac stress testing for valvular disease |
| **Reporting of search strategy should include** | |  |
| √ | Qualifications of searchers | The credentials of the two investigators JL and SB are indicated in the author list. Both are board-certified in internal medicine. |
| √ | Search strategy, including time period included in the synthesis and keywords | Time period: From October 1, 2005-March 1, 2015 in PubMed (which includes the MEDLINE database and other sources).  PubMed search strategy:  "stress test" OR exercise test[MeSH Terms] OR Cardiac Imaging Techniques[MeSH Terms] OR "thallium" OR sestamibi OR "technetium" OR MPI OR "myocardial perfusion" OR SPECT OR Myocardial Perfusion Imaging[MeSH Terms] OR Single Photon Emission Computed Tomography[MeSH Terms] OR echo OR echocardiography[MeSH Terms]) AND (approp* OR inapprop*) AND (English[lang]) AND ("2005/10/01"[PDAT] : “2015/3/1"[PDAT])  Additional articles identified through discussion with coauthors. |
| √ | Databases and registries searched | PubMed (which includes the MEDLINE database and other sources) |
| √ | Search software used, name and version, including special features | EndNote was used to manage citations and Microsoft Excel was used to merge retrieved citations and eliminate duplicates |
| √ | Use of hand searching | We did not perform hand-searching |
| √ | List of citations located and those excluded, including justifications | Details of the literature search are outlined in the flow chart and manuscript. Citation list is available upon request. |
| √ | Method of addressing articles published in languages other than English | We limited our search to articles published in the English language |
| √ | Method of handling abstracts and unpublished studies | We did not include abstracts or unpublished studies. |
| √ | Description of any contact with authors | Contacted Zachary Gertz, MD (Virginia Commonwealth University) regarding a question about an article he authored. |
| **Reporting of methods should include** | |  |
| √ | Description of relevance or appropriateness of studies assembled for assessing the hypothesis to be tested | Detailed inclusion and exclusion criteria are provided in the Methods section, along with a table of included studies. |
| √ | Rationale for the selection and coding of data | Data extracted from each of the studies on appropriateness rates of cardiac stress testing were relevant to the study population and exposure of interest. |
| √ | Assessment of confounding | Appropriateness rates were stratified by gender, physician specialty, and time period (typically pre-quality improvement intervention vs. post-quality improvement intervention) in some studies. |
| √ | Assessment of study quality, including blinding of quality assessors; stratification or regression on possible predictors of study results | No validated quality scales for assessments of AUC are available. We considered a study to be of adequate quality if authors described how they applied the AUC and appropriateness outcomes were reported. Meta-regression was also performed and the methods used can be found in the Methods, Statistical Analysis section. |
| √ | Assessment of heterogeneity | Heterogeneity of the studies was examined using Cochrane’s Q test of heterogeneity and I^2^ statistic that estimates the relative amount of variance attributable to between-study heterogeneity. |
| √ | Description of statistical methods in sufficient detail to be replicated | Description of methods of meta-analyses and sensitivity analyses are provided in detail in the Methods. |
| √ | Provision of appropriate tables and graphics | We included 1 flow chart, 4 forest plots of all studies, and 3 tables summarizing study characteristics and measures of stress testing appropriateness. |
| **Reporting of results should include** | |  |
| √ | Graph summarizing individual study estimates and overall estimate | Figures 2, 3, 4 and 5 |
| √ | Table giving descriptive information for each study included | Table 1 |
| √ | Results of sensitivity testing (e.g., subgroup analysis) | Included in Results section of manuscript. All appropriateness results stratified by AUC version and simple meta-regression performed to compare physician specialty appropriateness rates. |
| √ | Indication of statistical uncertainty of findings | We presented 95% confidence intervals for all summary estimates, I^2^ values, and sensitivity analyses |
| **Reporting of discussion should include** | |  |
| √ | Quantitative assessment of bias | Publication bias was assessed using funnel plots and the Egger test. A detailed description is provided in the Methods, Statistical Analysis section |
| √ | Justification for exclusion | Exclusion criteria were pre-defined and determined by how applicable reported measures were to outcomes of interest. |
| √ | Assessment of quality of included studies | All included studies were considered to be of adequate quality because all described how AUC were applied and reported at least one appropriateness outcome. |
| **Reporting of conclusions should include** | |  |
| √ | Consideration of alternative explanations for observed results | While several studies reported fairly similar appropriateness rates, it is important to note that appropriateness patterns in the community may differ from those in academic medical centers; most of our studies were from academic medical centers. In addition, the interpretation of AUC guidelines may vary by site. |
| √ | Generalization of the conclusions | Our results may not be generalizable to sites where practice patterns differ substantially from those in our studies. |
| √ | Guidelines for future research | Exploration of reasons for variation in appropriateness rates; Integration of appropriateness guidelines in both academic and community settings may be an effective approach to further optimizing the inappropriate/rarely appropriate use of cardiac stress testing and its associated costs and patient harms |
| √ | Disclosure of funding source | Dr. Ladapo's work is supported by a K23 Career Development Award (K23 HL116787) from the National Heart, Lung, and Blood Institute (NHLBI) and he serves as a consultant to CardioDx, Inc. Dr. Blecker’s work is supported by a K08 Career Development Award (K08 HS23683) from the Agency for Healthcare Research and Quality. |
